# Relationship Between Sleep and Quality of Life in Older Adults With Mild Cognitive Impairment

**DOI:** 10.1093/geroni/igaa057.2107

**Published:** 2020-12-16

**Authors:** Darina Petrovsky, Miranda Varasse, Nancy Hodgson

**Affiliations:** 1 University of Pennsylvania, Philadelphia, Pennsylvania, United States; 2 University of Pennsylvania, Cherry Hill, New Jersey, United States

## Abstract

Our objective was to examine the independent relationship between sleep characteristics and quality of life (QOL) in community-dwelling older adults with cognitive impairment. Objective sleep variables were derived from actigraphy and included total sleep time, wake after sleep onset (WASO), efficiency, and number of awakenings. Subjective sleep quality was measured using Pittsburgh Sleep Quality Index and daytime sleepiness was measured with the Epworth Sleepiness Scale. Caregiver reported QOL-AD was used for QOL. Analyses included Spearman’s correlation and multivariate linear regression. In bivariate analyses, QOL was significantly related to clinical dementia rating scale, sex, depression, daytime sleepiness, sleep quality, WASO, and number of awakenings. Controlling for depression, daytime sleepiness remained independently associated with QOL (β= -0.24; p= 0.03). In addition, number of awakenings trended towards significance (β= -0.13; p= 0.07). Results suggest daytime sleepiness and awakenings are associated with QOL in this population.

